# Supplementary material for: Structural basis for functional interactions in dimers of SLC26 transporters
Source: Nat Commun. 2019 May 2;10:2032. doi: 10.1038/s41467-019-10001-w (PMC6497670; doi:10.1038/s41467-019-10001-w)
Supplement: Supplementary file 1 — Supplementary Information [file 41467_2019_10001_MOESM1_ESM.pdf]

Structural basis for functional interactions in dimers of SLC26 transporters

Chang et al.

Supplementary Information

Supplementary Table 1. Spin labeling efficiency and the modulation depth for single-cysteine mutants of SLC26Dg

Position	spin labeling efficiency (%)	PELDOR modulation depth
SLC26Dg K353R1	74	12
SLC26Dg V367R1	88	28
SLC26Dg L385R1	81	26
SLC26Dg V129R1	100	30
SLC26Dg L144R1	97	
SLC26Dg W152R1	69	
SLC26Dg M154R1	80	
SLC26Dg I164R1	98	
SLC26Dg L168R1	100	
SLC26Dg V180R1	100	
SLC26Dg V188R1	100	
SLC26Dg L248R1	87	27
SLC26Dg M325R1	100	
SLC26Dg S338R1	91	

The MTSL labeling efficiency for SLC26Dg variants was determined in detergent micelles and the modulation depth value was determined from the PELDOR data obtained with proteoliposomes. The corresponding cw EPR spectra are shown in Supplementary Figure 1. For those positions for which the PELDOR data could be measured sufficiently long, the labeling efficiency and modulation depth values agree with each other. Position 353 seems to be an outlier with a slightly lower value, which may be due to an overestimation of the labeling efficiency or the loss of spin labels during reconstitution. A 10-15% error is estimated for the determination of the spin labeling efficiencies.

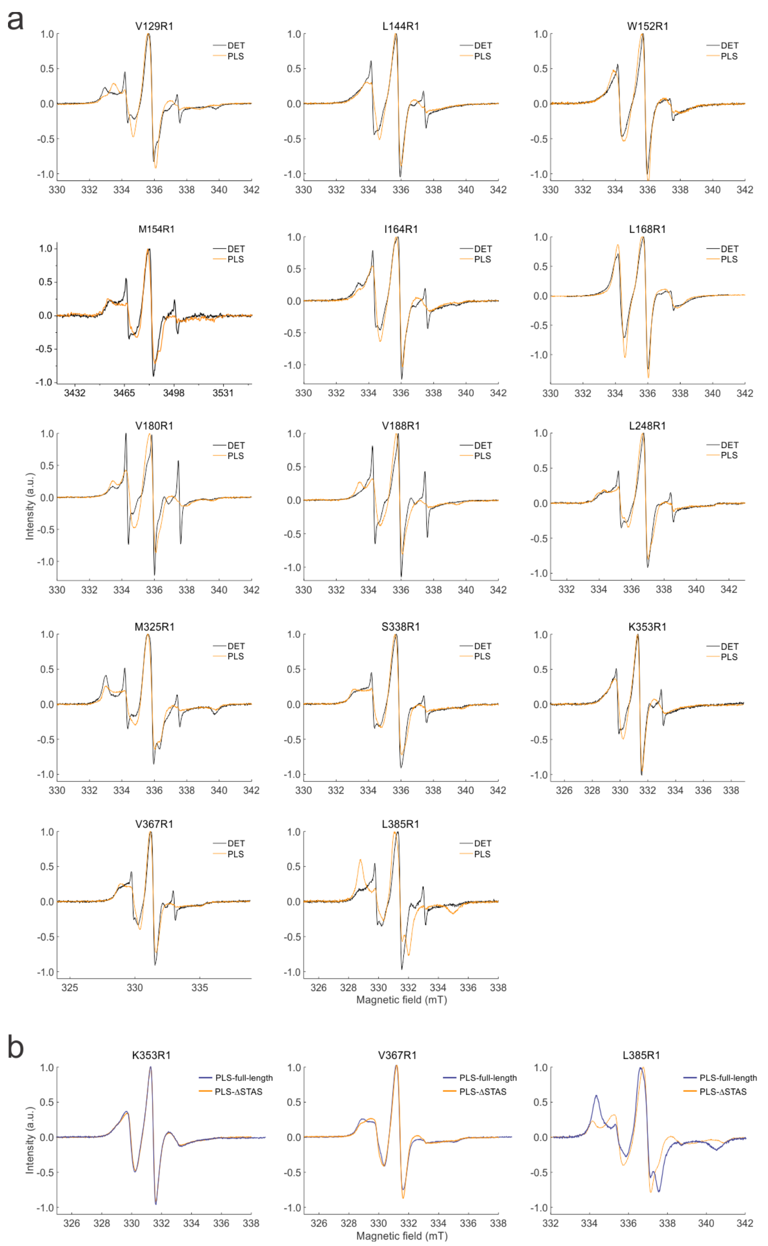

Supplementary Figure 1. Continuous-wave EPR spectra of spin-labeled SLC26Dg variants. (a) Comparison of cw-spectra of SLC26Dg mutants in detergent micelles (black trace) and in proteoliposomes (orange trace). **(b)** Comparison of cw-spectra of full-length SLC26Dg (blue trace) and SLC26Dg$^{\Delta STAS}$ (orange trace) mutants in proteoliposomes. Position L385R1 shows large changes in the spectrum upon STAS domain deletion, whereas the changes for positions K353R1 and V367R1 are comparably small. Accompanying PELDOR data is shown in Supplementary Figure 8. Spectra were acquired at room temperature. Normalized spectra are shown.

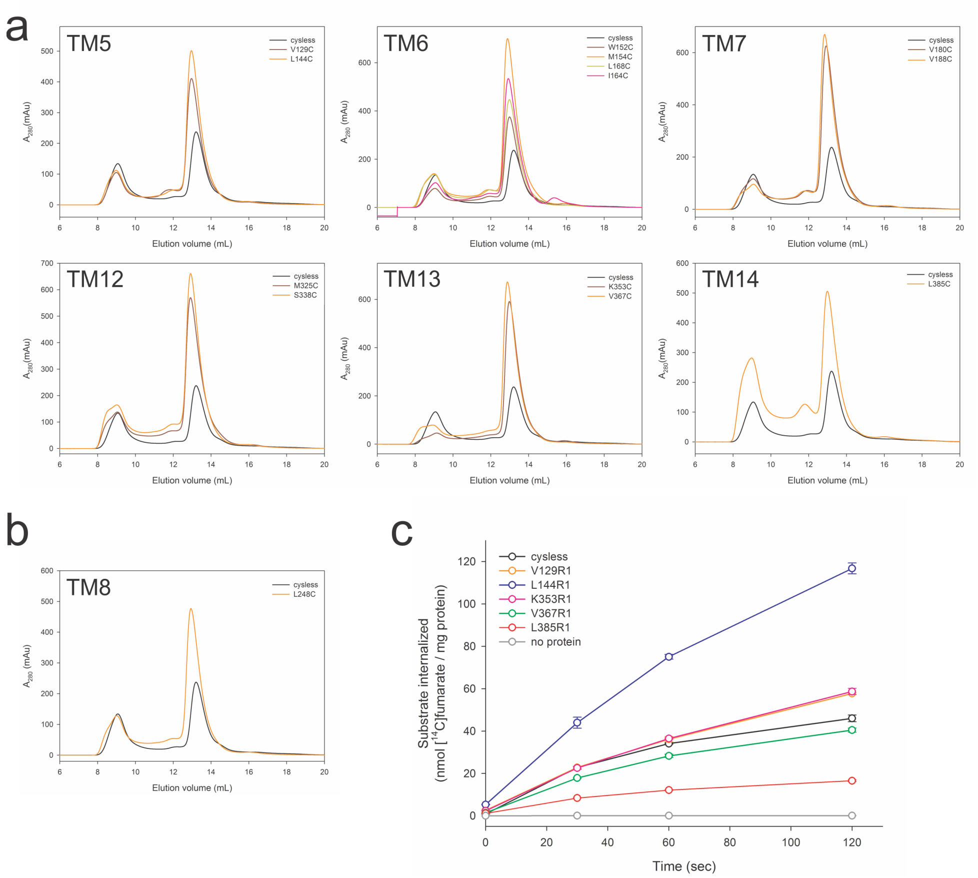

Supplementary Figure 2. Assessment of the folding state of single cysteine mutants of SLC26Dg. (a) Analysis of decylmaltoside-solubilized gate domain cysteine mutants of SLC26Dg by size exclusion chromatography. **(b)** Analysis of decylmaltoside-solubilized core domain mutant L248C by size exclusion chromatography. **(c)** Fumarate transport of spin-labeled SLC26Dg mutants in proteoliposomes. Substrate transport was normalized to the amount of membrane-inserted protein. Data represent mean and standard deviations of three technical replicates. Source data are provided as a Source Data file.

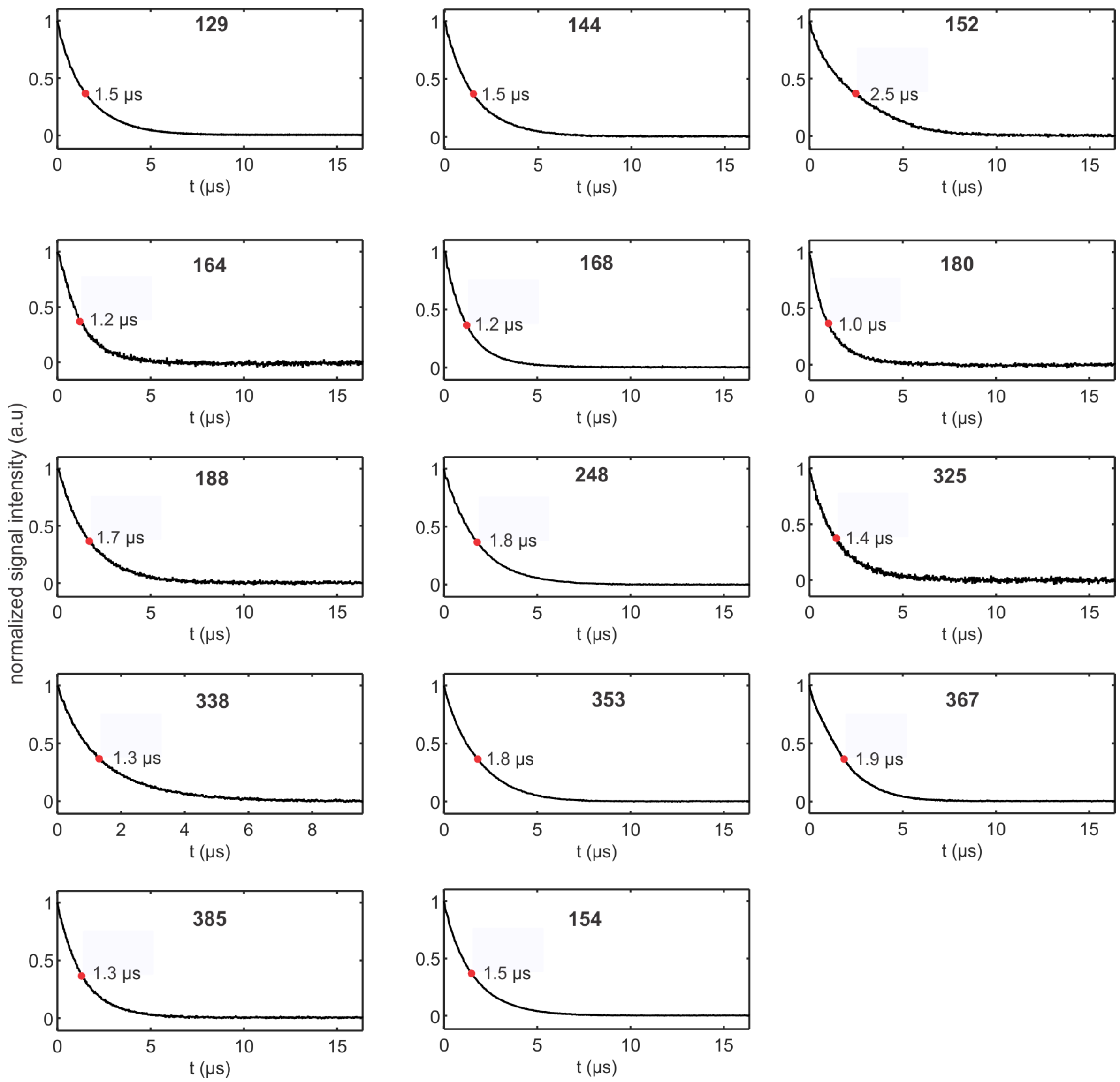

Supplementary Figure 3. Two-pulse echo decay traces for membrane-reconstituted spin-labeled SLC26Dg variants. The signal intensity was monitored as a function of the interpulse delay with steps of 8 ns increment, starting from an initial value between 200-400 ns. The time at which the signal intensity drops to 1/e (indicated with the red dot) represents the T_M.

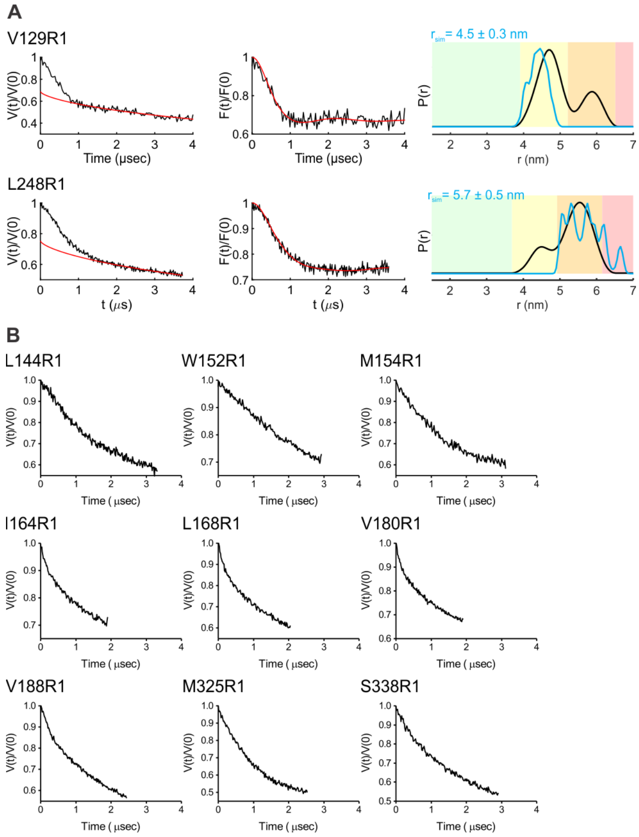

Supplementary Figure 4. Overview of PELDOR measurements on membrane-reconstituted SLC26Dg. (a) All the positions revealed a rather short T_M (Supplementary Figure 3). Original data (left), background corrected form factor (middle, in black) overlaid with the fit from Tikhonov regularization (in red), and the P(r) (right) for positions V129R1 located in TM5 (gate domain) and L248R1 located in TM8 (core domain). Background coloring of the experimental P(r) (black trace) indicates the uncertainty for the mean distance, width, and the shape of the P(r) due to the limited time window for dipolar evolution. In the green region, the mean, width, and the shape are reliable, in the yellow region the mean and the width are reliable, and in the orange region only the mean is reliable. Distances in the red region are unreliable. The corresponding simulations for P(r) (blue trace) performed on the dimer model are also shown. Overall, the experimental P(r)s are in agreement with the simulations and indicate some additional flexibility in proteoliposomes as compared to the static dimer model. **(b)** Original data for positions on TM5 (144), TM6 (152, 154, 164, and 168), TM7 (180 and 188), and TM12 (325 and 338). Simulations for these positions predict mean distances between 6.3-10.6 nm. Although the data indicate the presence of such long distances, time traces of sufficient length to determine those distances could not be acquired owing to short T_M.

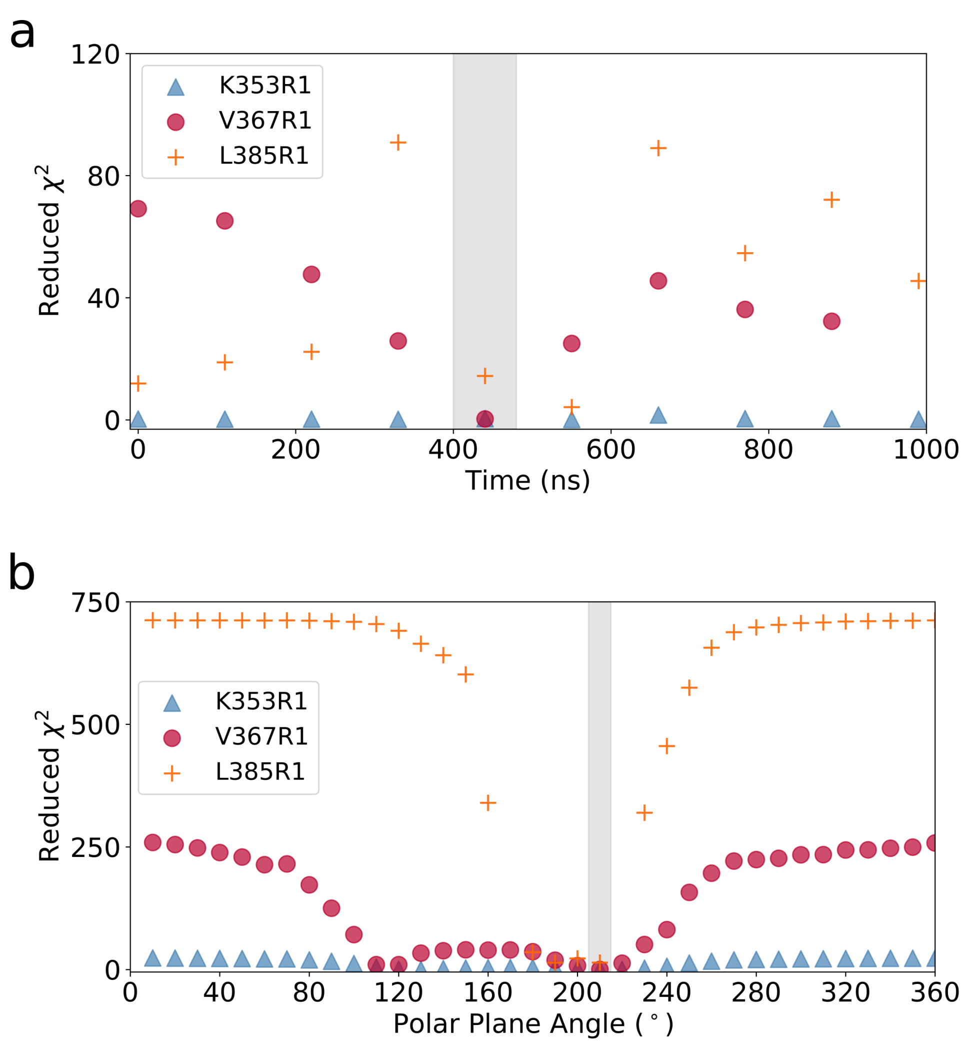

Supplementary Figure 5. Rigid-body docking of SLC26Dg dimer. (a) Selection of the SLC26Dg conformation to be used for rigid-body docking of the dimer from MD simulation of a membrane-embedded SLC26Dg monomer starting from the crystal structure. The consistency of potential SLC26Dg dimers with experimental PELDOR signals is quantified by the reduced χ^2 as a function of MD time for each spin-label position K353R1 (blue), V367R1 (red), and L385R1 (orange). The snapshot at 440 ns (grey bar) was taken for further analysis of the SLC26Dg dimer. **(b)** Uniqueness of rigid-body docking solution for SLC26Dg dimer. The reduced χ^2 of measured and calculated PELDOR signals for the three label sites is plotted as a function of the polar plane angle defining the orientation of the second protomer in the membrane plane under C2 symmetry. At an angle of 210 degrees (grey bar), all three PELDOR signals are reproduced, thereby defining the orientation taken for further analysis.

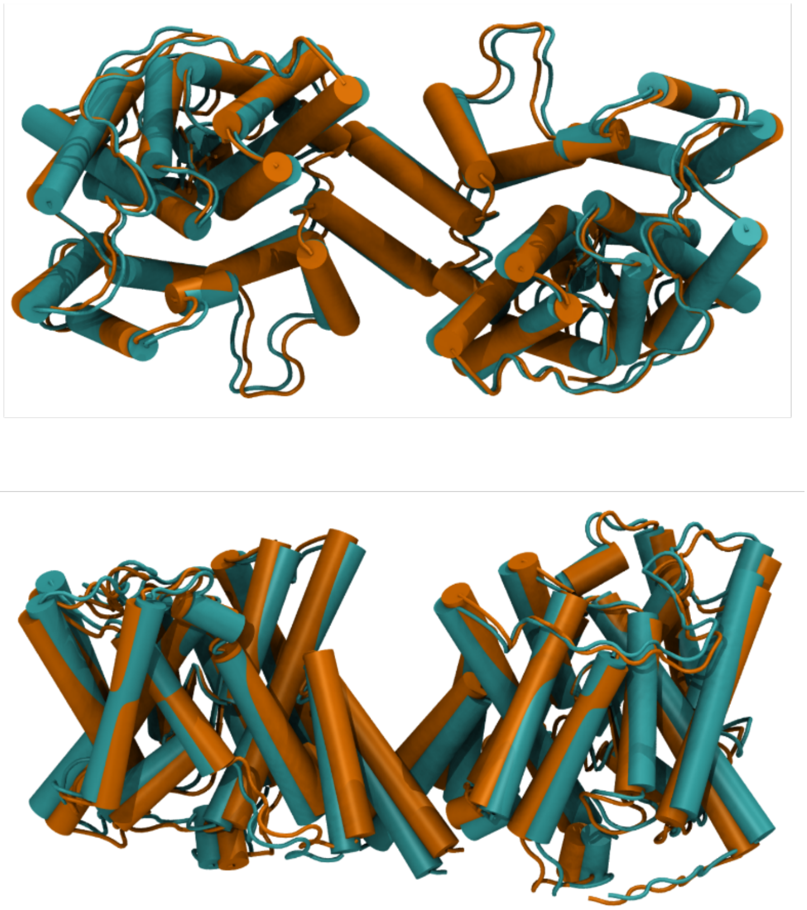

Supplementary Figure 6. Comparison of SLC26Dg dimer models obtained via independent approaches. Superimposition of the models generated using MMMDock (orange) based on distance distributions P(r) and the model based on form factors calculated for MD dimer structures using BioEn rotamer refinement (teal). View from the extracellular side (top) and in the plane of the membrane (bottom).

Neither model has backbone clashes. The BioEn model does not have any side chain clashes either. The two models align to each other with a global backbone root mean square deviation (RMSD) of 1.7 Å. The MMMDock model exhibits two side chain clashes as well as contacts between some of the side chain atoms. The form factor based structure which is further minimized with MD simulation is the structure discussed in the manuscript. Despite the small differences between the structures, the fact that a very similar structure is obtained via two independent approaches largely excludes any bias by the fitting routines for the dimer structure.

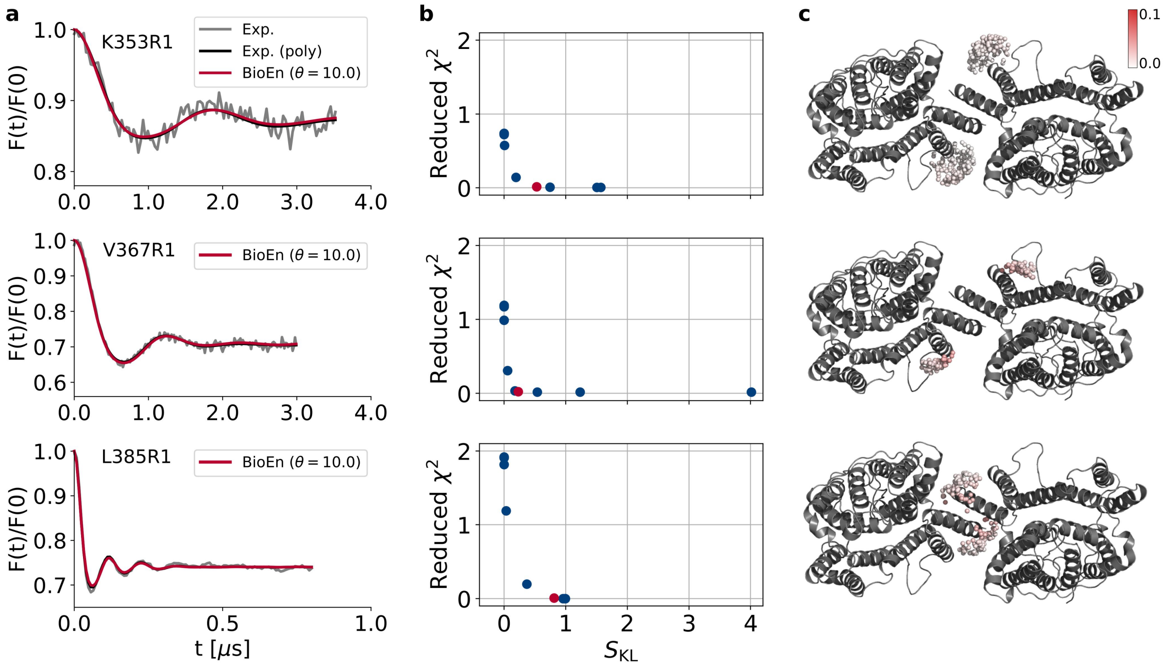

Supplementary Figure 7. BioEn spin-label rotamer refinement using experimental PELDOR signals reveal weights of rotamer states. (a) For each labeled position (K353R1, V367R1, L385R1), the experimental PELDOR signals (gray), a polynomial fit to the experimental signals (black) and the calculated BioEn PELDOR traces after spin-label ensemble refinement are shown. Within the BioEn procedure, for each calculated PELDOR signal the result of the optimal confidence value θ_i is shown. **(b)** L-curve analysis for the three PELDOR measurements. Reduced χ^2 is plotted as a function of the Kullback-Leibler relative entropy S_{KL} for $\theta = 10^6$ to $\theta = 10^{-1}$ (left to right; blue), with θ decreased stepwise by factors of 10. The rightmost point is the result of a regular χ^2 minimization ($\theta = 0$). Red circles indicate the results for $\theta = 10^1$, which is the value of θ chosen for further analyses. **(c)** Spin-label centers for SLC26Dg dimer (white to red spheres). Darker colors indicate higher relative weights (see scale bar).

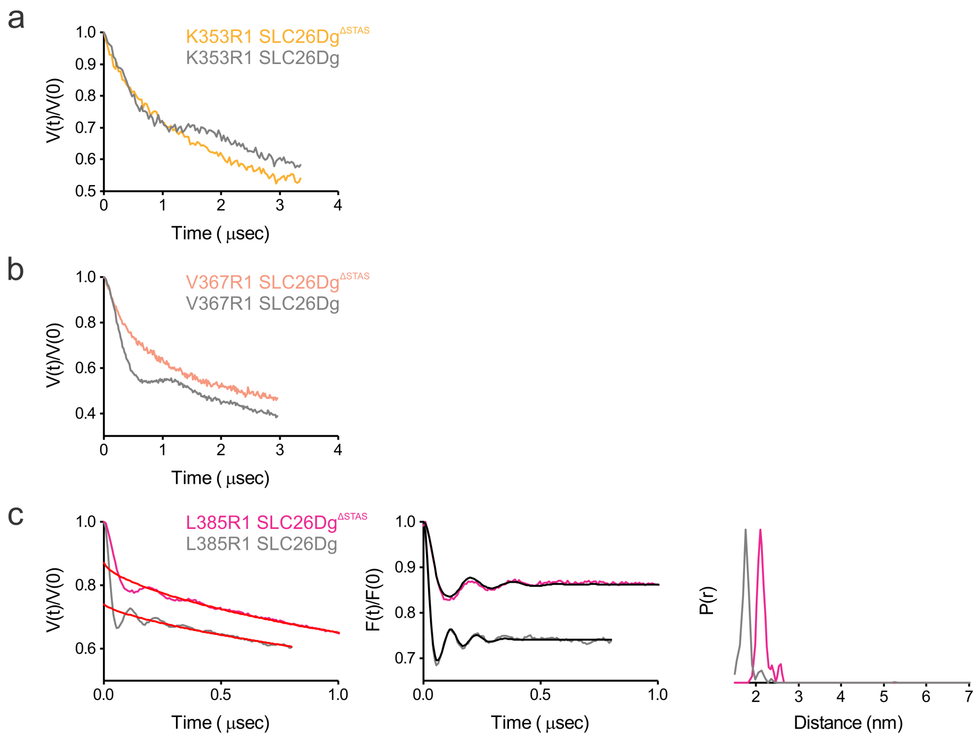

Supplementary Figure 8. Interspin distances in the SLC26Dg$^{\Delta STAS}$ dimer. Comparison of PELDOR data for membrane-reconstituted full-length (grey) and STAS deletion mutants of SLC26Dg as indicated. **(a-b)** For positions K353R1 and V367R1, which are located in TM13, deletion of the STAS domain leads to faster damping of V(t)/V(0), suggesting that either rearrangement of the spin label rotamers or an increased flexibility at these positions underlies these changes. The limited time window of the dipolar evolution does not allow to discriminate between these potential causes. **(c)** For L385R1 located in TM14, a small shift of the mean distance is observed, that, given the narrow distance distribution, rather suggests a rearrangement of the MTSSL rotamers than a physical separation of the protomers. Altogether, the data show that STAS deletion does not prevent dimerization.

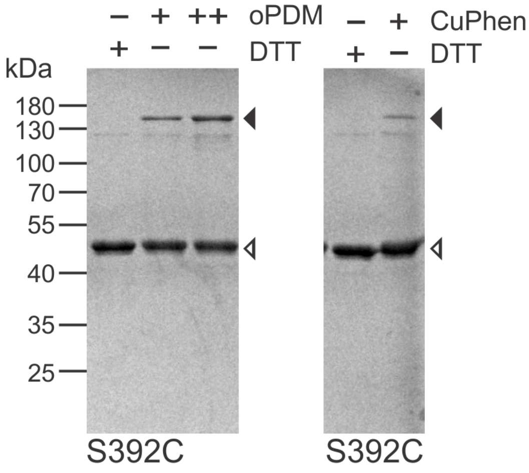

Supplementary Figure 9. Cysteine crosslinking of SLC26Dg(S392C) in proteoliposomes. SLC26Dg(S392C) was purified and membrane-reconstituted in the presence of reducing reagents. Crosslinking was initiated by the addition of 50 µM (single plus) or 500 µM (double plus) N,N'-1,2-phenylenedimaleimide (oPDM, left panel) or Cu-phenanthroline (right panel). Samples were incubated for 20 min at room temperature and subsequently analyzed by SDS-PAGE and visualized by coomassie staining. Black and white arrows indicate dimeric and monomeric SLC26Dg, respectively.

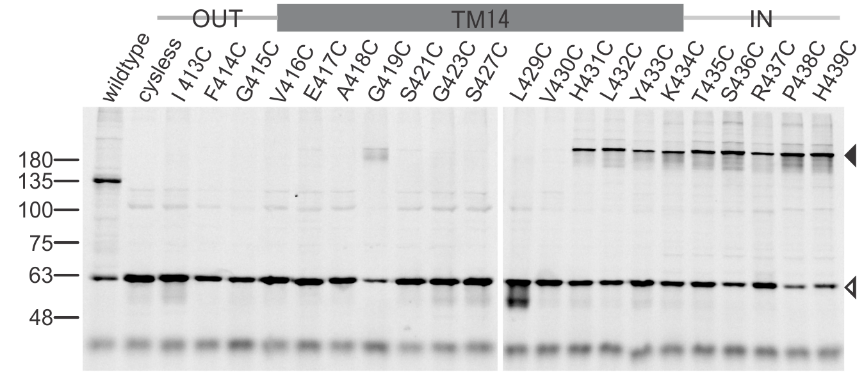

Supplementary Figure 10. Oxidative cysteine crosslinking between TM14 of SLC26Si. In gel GFP fluorescence analysis of disrupted *E. coli* cells expressing single cysteine variants of SLC26Si fused to superfolder-GFP. The approximate boundaries of TM14 are indicated above. At the center of TM14, covering G419-L429, every second residue was analyzed. Following oxidative crosslinking, samples were analyzed by non-reducing SDS-PAGE. Wildtype (holding a native cysteine following the C-terminal STAS domain) and cysless represent positive and negative controls, respectively. Black and white arrows indicate dimeric and monomeric SLC26Si, respectively.

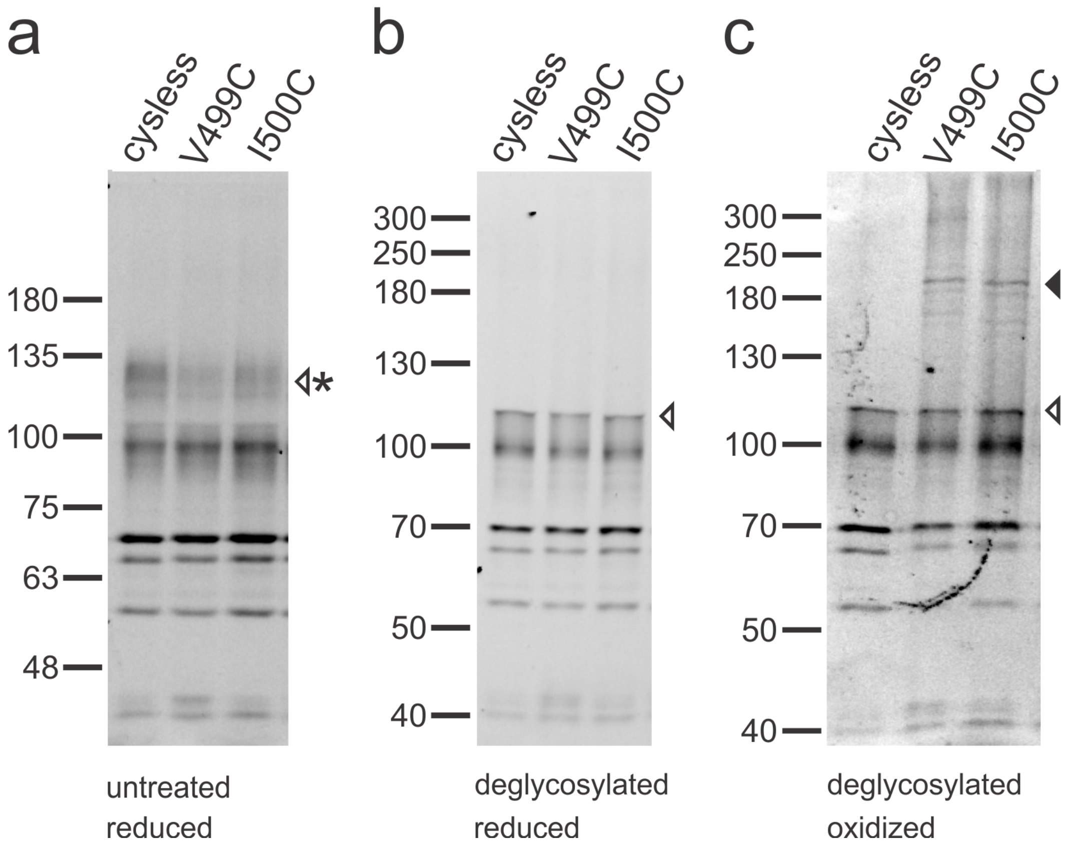

Supplementary Figure 11. Oxidative crosslinking between TM14 of rat prestin. In gel GFP fluorescence analysis of CHO membrane vesicles holding cysteine variants of rat prestin fused to eGFP. V499 and I500 are located at the cytoplasmic end of TM14. Samples were analyzed **(a)** prior, or **(b)** following deglycosylation by reducing SDS-PAGE, or **(c)** following deglycosylation and oxidative crosslinking by non-reducing SDS-PAGE. Black and white arrows indicate dimeric and monomeric rat prestin, respectively. The asterisk indicates glycosylated rat prestin. Lower molecular weight bands represent mostly non-glycosylated, proteolytic fragments of rat prestin.

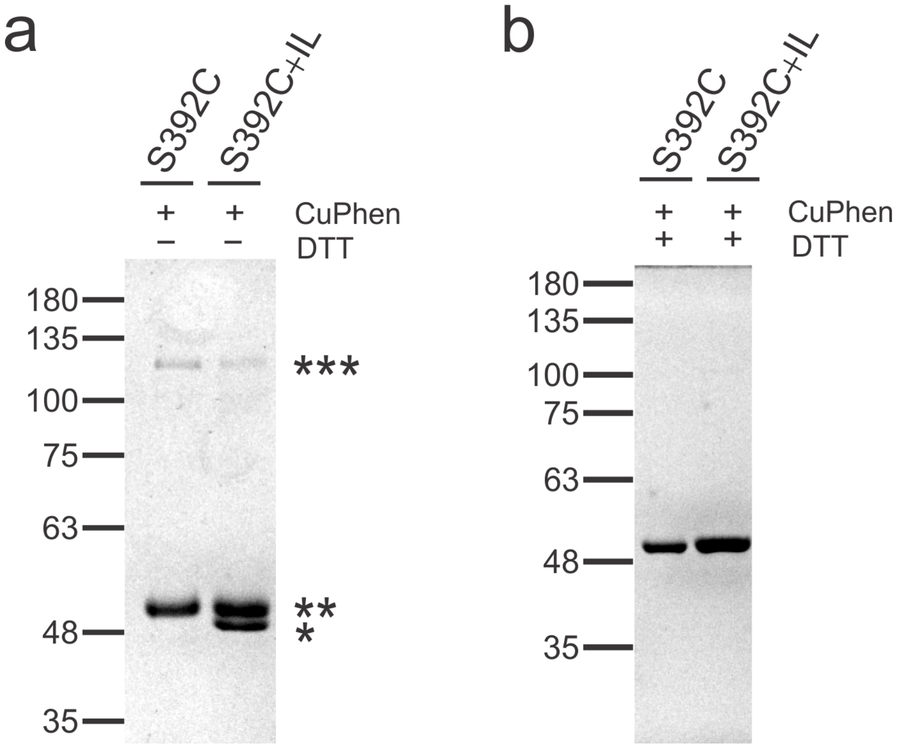

Supplementary Figure 12. Stochastic formation of heterodimers upon membrane-reconstitution of monomers. (a) SLC26Dg(S392C) (100%) and a 50:50 mixture of SLC26Dg(S392C) : SLC26Dg-IL were membrane-reconstituted and following oxidative crosslinking, analyzed by non-reducing SDS-PAGE and visualized by coomassie staining. Similar amounts of S392C are loaded in both lanes. The triple, double and single asterisk indicate the S392C-S392C homodimer, the monomeric S392C and the crosslinked IL mutant, respectively. The decrease in the amount of crosslinked S392C upon addition of IL indicates the formation of S392C-IL heterodimers and suggests that protomers combine to dimers in a random manner. **(b)** Reducing SDS-PAGE analysis of samples from panel (a) visualized by coomassie staining.

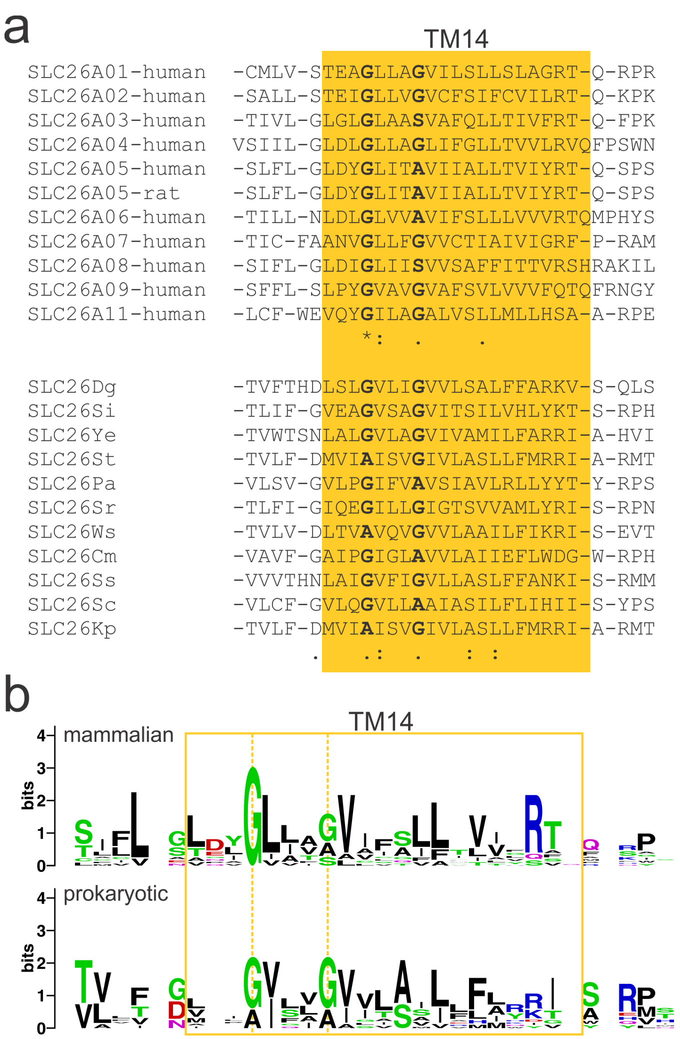

Supplementary Figure 13. Sequence alignment of TM14 from mammalian and prokaryotic SLC26 proteins. (a) Amino acid alignments performed using AlignMe [1] with SLC26Dg as reference sequence. Prokaryotic SLC26 proteins analyzed are from *Deinococcus geothermalis* (Dg), *Sulfitobacter indolifex* (Si), *Yersinia enterocolitica* (Ye), *Salmonella typhimurium* (St), *Pseudomonas aeruginosa* (Pa), *Salinibacter ruber* (Sr), *Wolinella succinogenes* (Ws), Cupriavidus metallidurans (Cm), *Shewanella sediminis* (Ss), *Synechocystis* sp. (Sc), and *Klebsiella pneumonia* (Kp). The selected mammalian and prokaryotic SLC26 proteins have an average amino acid identity between each other of 31% and 32%, respectively. Gly, Ala, and Ser residues part of the GxxxG-like motif are indicated in bold. **(b)** Sequence logo [2] representation of the alignments in (a).

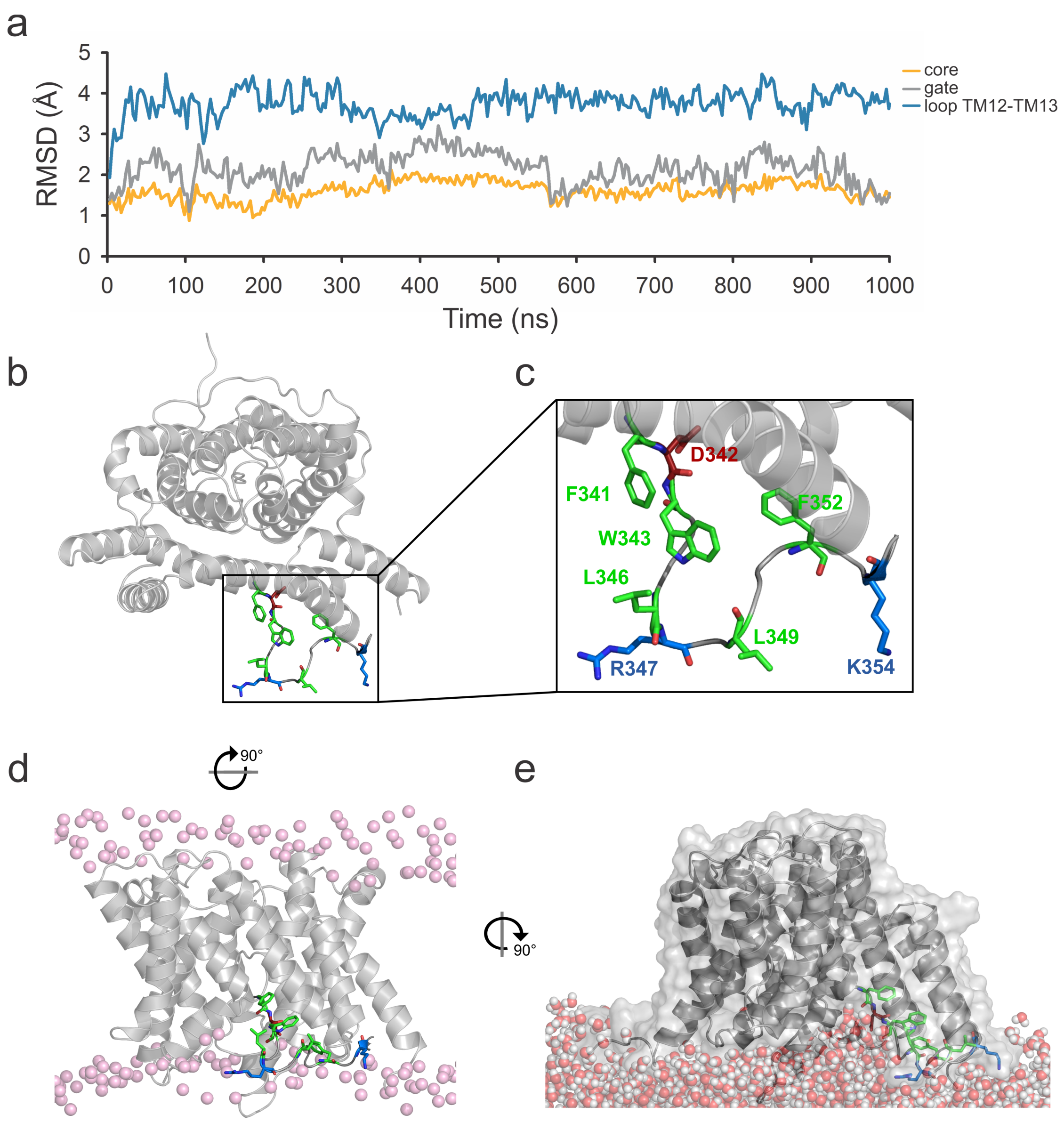

Supplementary Figure 14. Reduced flexibility of the cytoplasmic loop connecting TM12 and 13 observed in the MD simulation. (a) C_{α}-atom root-mean-squared-distance values of the core, gate, and the cytoplasmic loop connecting TM12 and 13 relative to the monomer crystal structure as a function of MD time (1 µs). **(b)** Top-view of SLC26Dg is shown with highlighted residues: R347 and K354 (blue), D342 (red) and hydrophobic residues F341, W343, L346, L349, F352 (green). **(c)** Detailed view on the residues of the respective. **(d)** Side-view of SLC26DG with residues highlighted and lipid headgroups (phosphorus atoms) shown as spheres. **(e)**. Turned side-view to indicate funnel of water molecules towards the binding site.

Supplementary References

1. Stamm M, Staritzbichler R, Khafizov K, Forrest LR. AlignMe--a membrane protein sequence alignment web server. *Nucleic Acids Res* **42**, W246-251 (2014).

2. Crooks GE, Hon G, Chandonia JM, Brenner SE. WebLogo: a sequence logo generator. *Genome Res* **14**, 1188-1190 (2004).
