# Supplementary material for: Structural basis for functional interactions in dimers of SLC26 transporters
Source: Nat Commun. 2019 May 2;10:2032. doi: 10.1038/s41467-019-10001-w (PMC6497670; doi:10.1038/s41467-019-10001-w)
Supplement: Supplementary file 2 — Supplementary Data 1 [file 41467_2019_10001_MOESM2_ESM.pdf]

Structural basis for functional interactions in dimers of SLC26 transporters

Chang et al.

Supplementary Data 1 - Primers used for mutagenesis

Name	Sequence (5' - 3')
pINIT_F	GAGTAGGACAAATCCGC
pINIT_R	TGGCAGTTTATGGCGGGCGT
SLC26Dg_I45C_F	GCTTTCTCCATCTGCGCGGGCGTCGATCCGCAG
SLC26Dg_I45C_R	ATCGACGCCCGCGCAGATGGAGAAAGCGATTGC
SLC26Dg_A142C_F	CTGATCTTTATGTGCCAGCTCCCCCAGTTCGTA
SLC26Dg_A142C_R	CTGGGGGAGCTGGCACATAAAGATCAGGATGGC
SLC26Dg_V129C_F	CGCAGCGTCATGTGCGGCTTCGTGAATGCGCTG
SLC26Dg_V129C_R	ATTCACGAAGCCGCACATGACGCTGCGCGGCAC
SLC26Dg_L144C_F	TTTATGGCGCAGTGCCCCCAGTTCGTAGGTGCG
SLC26Dg_L144C_R	TACGAACTGGGGGCACTGCGCCATAAAGATCAG
SLC26Dg_W152C_F	GTAGGTGCGAACTGCCAGATGTACGCCATGGTC
SLC26Dg_W152C_R	GGCGTACATCTGGCAGTTCGCACCTACGAACTG
SLC26Dg_M154C_F	GCGAACTGGCAGTGCTACGCCATGGTCGCCGCT
SLC26Dg_M154C_R	GACCATGGCGTAGCACTGCCAGTTCGCACCTAC
SLC26Dg_I164C_F	GCTGGTCTGGCCTGCATCTACCTCCTCCCACTC
SLC26Dg_I164C_R	GAGGAGGTAGATGCAGGCCAGACCAGCGGCGAC
SLC26Dg_L168C_F	ATCATCTACCTCTGCCCACTCGTGTTCAAAGCT
SLC26Dg_L168C_R	GAACACGAGTGGGCAGAGGTAGATGATGGCCAG
SLC26Dg_V180C_F	CCCAGTGCGCTGTGCGCCATCGTCGTCCTCACG
SLC26Dg_V180C_R	GACGACGATGGCGCACAGCGCACTGGGCATAGC
SLC26Dg_V188C_F	GTCCTCACGGTGTGCGCGGTGGTGACCGGCGCG
SLC26Dg_V188C_R	GGTCACCACCGCGCACACCGTGAGGACGACGAT
SLC26Dg_L248C_F	CTGACCGCGCAGTGCATCGACGAACGAACCGAT
SLC26Dg_L248C_R	TCGTTCGTCGATGCACTGCGCGGTCAGCAGACT
SLC26Dg_M325C_F	GTGCAGATCCCGTGCGCGGCGCTGGTGGCCGTG
SLC26Dg_M325C_R	CACCAGCGCCGCGCACGGGATCTGCACCAGCAG
SLC26Dg_S338C_F	GTCGTGGCGATCTGCACCTTTGATTGGGGGAGC
SLC26Dg_S338C_R	CCAATCAAAGGTGCAGATCGCCACGACCATCAT
SLC26Dg_K353C_F	ACGGTCTTCCCGTGCGGGGAGACCGTCGTCATG
SLC26Dg_K353C_R	GACGGTCTCCCCGCACGGGAAGACCGTCAGCGT
SLC26Dg_V367C_F	GTCGCCGTGACGTGCTTCACACATGACCTGAGT
SLC26Dg_V367C_R	GTCATGTGTGAAGCACGTCACGGCGACCGTGGC
SLC26Dg_T369C_F	GCCGTGACGGTGTTCTGCCATGACCTGAGTCTG
SLC26Dg_T369C_R	CAGACTCAGGTCATGGCAGAACACCGTCACGGC
SLC26Dg_H370C_F	GTGACGGTGTTCACATGCGACCTGAGTCTGGGT
SLC26Dg_H370C_R	ACCCAGACTCAGGTCGCATGTGAACACCGTCAC
SLC26Dg_D371C_F	ACGGTGTTCACACATTGCCTGAGTCTGGGTGTC

SLC26Dg_D371C_R	GACACCCAGACTCAGGCAATGTGTGAACACCGT
SLC26Dg_L372C_F	GTGTTCACACATGACTGCAGTCTGGGTGTCTTG
SLC26Dg_L372C_R	CAAGACACCCAGACTGCAGTCATGTGTGAACAC
SLC26Dg_S373C_F	TTCACACATGACCTGTGCCTGGGTGTCTTGATC
SLC26Dg_S373C_R	GATCAAGACACCCAGGCACAGGTCATGTGTGAA
SLC26Dg_L374C_F	CATGACCTGAGTTGCGGTGTCTTGATCGGTGTG
SLC26Dg_L374C_R	GATCAAGACACCGCAACTCAGGTCATGTGTGAA
SLC26Dg_G375C_F	CATGACCTGAGTCTGTGCGTCTTGATCGGTGTG
SLC26Dg_G375C_R	CACACCGATCAAGACGCACAGACTCAGGTCATG
SLC26Dg_V376C_F	GACCTGAGTCTGGGTTGCTTGATCGGTGTGGTG
SLC26Dg_V376C_R	CACCACACCGATCAAGCAACCCAGACTCAGGTC
SLC26Dg_L377C_F	CTGAGTCTGGGTGTCTGCATCGGTGTGGTGCTC
SLC26Dg_L377C_R	GAGCACCACACCGATGCAGACACCCAGACTCAG
SLC26Dg_I378C_F	AGTCTGGGTGTCTTGTGCGGTGTGGTGCTCAGC
SLC26Dg_I378C_R	GCTGAGCACCACACCGCACAAGACACCCAGACT
SLC26Dg_G379C_F	CTGGGTGTCTTGATCTGCGTGGTGCTCAGCGCG
SLC26Dg_G379C_R	CGCGCTGAGCACCACGCAGATCAAGACACCCAG
SLC26Dg_V380C_F	GGTGTCTTGATCGGTTGCGTGCTCAGCGCGTTG
SLC26Dg_V380C_R	CAACGCGCTGAGCACGCAACCGATCAAGACACC
SLC26Dg_V381C_F	GTCTTGATCGGTGTGTGCCTCAGCGCGTTGTTC
SLC26Dg_V381C_R	GAACAACGCGCTGAGGCACACACCGATCAAGAC
SLC26Dg_L382C_F	TTGATCGGTGTGGTGTGCAGCGCGTTGTTCTTC
SLC26Dg_L382C_R	GAAGAACAACGCGCTGCACACCACACCGATCAA
SLC26Dg_S383C_F	ATCGGTGTGGTGCTCTGCGCGTTGTTCTTCGCT
SLC26Dg_S383C_R	AGCGAAGAACAACGCGCAGAGCACCACACCGAT
SLC26Dg_A384C_F	GGTGTGGTGCTCAGCTGCTTGTTCTTCGCTCGG
SLC26Dg_A384C_R	CCGAGCGAAGAACAAGCAGCTGAGCACCACACC
SLC26Dg_L385C_F	GTGCTCAGCGCGTGCTTCTTCGCTCGGAAGGTG
SLC26Dg_L385C_R	CCGAGCGAAGAAGCACGCGCTGAGCACCACACC
SLC26Dg_F386C_F	GTGCTCAGCGCGTTGTGCTTCGCTCGGAAGGTG
SLC26Dg_F386C_R	CACCTTCCGAGCGAAGCACAACGCGCTGAGCAC
SLC26Dg_F387C_F	CTCAGCGCGTTGTTCTGCGCTCGGAAGGTGTCG
SLC26Dg_F387C_R	CGACACCTTCCGAGCGCAGAACAACGCGCTGAG
SLC26Dg_A388C_F	AGCGCGTTGTTCTTCTGCCGGAAGGTGTCGCAA
SLC26Dg_A388C_R	TTGCGACACCTTCCGGCAGAAGAACAACGCGCT
SLC26Dg_R389C_F	GCGTTGTTCTTCGCTTGCAAGGTGTCGCAACTC
SLC26Dg_R389C_R	GAGTTGCGACACCTTGCAAGCGAAGAACAACGC
SLC26Dg_K390C_F	TTGTTCTTCGCTCGGTGCGTGTCGCAACTCTCG
SLC26Dg_K390C_R	CGAGAGTTGCGACACGCACCGAGCGAAGAACAA

SLC26Dg_V391C_F	TTCTTCGCTCGGAAGTGCTCGCAACTCTCGCAG
SLC26Dg_V391C_R	CTGCGAGAGTTGCGAGCACTTCCGAGCGAAGAA
SLC26Dg_S392C_F	GCTCGGAAGGTGTGCCAACTCTCGCAGGTGACG
SLC26Dg_S392C_R	CTGCGAGAGTTGGCACACCTTCCGAGCGAAGAA
SLC26Dg_Q393C_F	GCTCGGAAGGTGTCGTGCCTCTCGCAGGTGACG
SLC26Dg_Q393C_R	CGTCACCTGCGAGAGGCACGACACCTTCCGAGC
SLC26Dg_L394C_F	CGGAAGGTGTCGCAATGCTCGCAGGTGACGCCG
SLC26Dg_L394C_R	CGGCGTCACCTGCGAGCATTGCGACACCTTCCG
SLC26Dg_S395C_F	AAGGTGTCGCAACTCTGCCAGGTGACGCCGGTG
SLC26Dg_S395C_R	CACCGGCGTCACCTGGCAGAGTTGCGACACCTT
SLC26Si_C567S_F	CATGAAGCTGTTAGCGCCTTAGCTAAGCAGCAC
SLC26Si_C567S_R	CTTAGCTAAGGCGCTAACAGCTTCATGCTGGCT
SLC26Si_I413C_F	ATCCTGCTCACGCTGTGCTTCGGCGTTGAAGCG
SLC26Si_I413C_R	CGCTTCAACGCCGAAGCACAGCGTGAGCAGGAT
SLC26Si_F414C_F	CTGCTCACGCTGATTTGCGGCGTTGAAGCGGGG
SLC26Si_F414C_R	CCCCGCTTCAACGCCGCAAATCAGCGTGAGCAG
SLC26Si_G415C_F	CTCACGCTGATTTTCTGCGTTGAAGCGGGGGTG
SLC26Si_G415C_R	CACCCCCGCTTCAACGCAGAAAATCAGCGTGAG
SLC26Si_V416C_F	ACGCTGATTTTCGGCTGCGAAGCGGGGGTGAGC
SLC26Si_V416C_R	GCTCACCCCCGCTTCGCAGCCGAAAATCAGCGT
SLC26Si_E417C_F	CTGATTTTCGGCGTTTGCGCGGGGGTGAGCGCG
SLC26Si_E417C_R	CGCGCTCACCCCCGCGCAAACGCCGAAAATCAG
SLC26Si_A418C_F	ATTTTCGGCGTTGAATGCGGGGTGAGCGCGGGG
SLC26Si_A418C_R	CCCCGCGCTCACCCCGCATTCAACGCCGAAAAT
SLC26Si_G419C_F	TTCGGCGTTGAAGCGTGCGTGAGCGCGGGGGTC
SLC26Si_G419C_R	GACCCCCGCGCTCACGCACGCTTCAACGCCGAA
SLC26Si_S421C_F	GTTGAAGCGGGGGTGTGCGCGGGGGTCATCACC
SLC26Si_S421C_R	GGTGATGACCCCCGCGCACACCCCCGCTTCAAC
SLC26Si_G423C_F	GCGGGGGTGAGCGCGTGCGTCATCACCTCGATC
SLC26Si_G423C_R	GATCGAGGTGATGACGCACGCGCTCACCCCCGC
SLC26Si_S427C_F	GCGGGGGTCATCACCTGCATCCTGGTCCATCTT
SLC26Si_S427C_R	AAGATGGACCAGGATGCAGGTGATGACCCCCGC
SLC26Si_L429C_F	GTCATCACCTCGATCTGCGTCCATCTTTATAAG
SLC26Si_L429C_R	CTTATAAAGATGGACGCAGATCGAGGTGATGAC
SLC26Si_V430C_F	ATCACCTCGATCCTGTGCCATCTTTATAAGACC
SLC26Si_V430C_R	GGTCTTATAAAGATGGCACAGGATCGAGGTGAT
SLC26Si_H431C_F	ACCTCGATCCTGGTCTGCCTTTATAAGACCTCG
SLC26Si_H431C_R	CGAGGTCTTATAAAGGCAGACCAGGATCGAGGT
SLC26Si_L432C_F	TCGATCCTGGTCCATTGCTATAAGACCTCGCGC

SLC26Si_L432C_R	GCGCGAGGTCTTATAGCAATGGACCAGGATCGA
SLC26Si_Y433C_F	ATCCTGGTCCATCTTTGCAAGACCTCGCGCCCG
SLC26Si_Y433C_R	CGGGCGCGAGGTCTTGCAAAGATGGACCAGGAT
SLC26Si_K434C_F	CTGGTCCATCTTTATTGCACCTCGCGCCCGCAT
SLC26Si_K434C_R	ATGCGGGCGCGAGGTGCAATAAAGATGGACCAG
SLC26Si_T435C_F	GTCCATCTTTATAAGTGCTCGCGCCCGCATATG
SLC26Si_T435C_R	CATATGCGGGCGCGAGCACTTATAAAGATGGAC
SLC26Si_S436C_F	CATCTTTATAAGACCTGCCGCCCGCATATGGCT
SLC26Si_S436C_R	AGCCATATGCGGGCGGCAGGTCTTATAAAGATG
SLC26Si_R437C_F	CTTTATAAGACCTCGTGCCCGCATATGGCTGTG
SLC26Si_R437C_R	CACAGCCATATGCGGGCACGAGGTCTTATAAAG
SLC26Si_P438C_F	TATAAGACCTCGCGCTGCCATATGGCTGTGGTG
SLC26Si_P438C_R	CACCACAGCCATATGGCAGCGCGAGGTCTTATA
SLC26Si_H439C_F	AAGACCTCGCGCCCGTGCATGGCTGTGGTGGGC
SLC26Si_H439C_R	GCCCACCACAGCCATGCACGGGCGCGAGGTCTT
ratprestin_F	ATATATGCTCTTCTAGTCTGCTGTGATCATTGCTCTGCTCACTG
ratprestin_R	TATATAGCTCTTCATGCGTGGCGACCGGTGGATCCCGGGCCCGC
ratprestin_V499C_F	GCTCTGCTCACTTGCATTTACAGAACACAGAGT
ratprestin_V499C_R	TGTTCTGTAAATGCAAGTGAGCAGAGCAATGAT
ratprestin_I500C_F	CTGCTCACTGTGTGCTACAGAACACAGAGTCCG
ratprestin_I500C_R	CTGTGTTCTGTAGCACACAGTGAGCAGAGCAAT

All primers are depicted in 5’ to 3’ direction. Forward and reverse primers are indicated by “F” and “R”, respectively.
