# Supplementary material for: Structural basis for functional interactions in dimers of SLC26 transporters
Source: Nat Commun. 2019 May 2;10:2032. doi: 10.1038/s41467-019-10001-w (PMC6497670; doi:10.1038/s41467-019-10001-w)
Supplement: Supplementary file 4 — Description of Additional Supplementary Files [file 41467_2019_10001_MOESM4_ESM.docx]

Title: Supplementary Data 1

Description: Primers used for mutagenesis
